# Right‐Sided Bilothorax With Secondary Empyema: A Rare Thoracic Manifestation

**DOI:** 10.1155/crpu/3080834

**Published:** 2026-02-27

**Authors:** Aris Sudarwoko, Anna Febriani, Donny Ardika Novananda

**Affiliations:** ^1^ Pulmonology and Respiratory Medicine Department, Faculty of Medicine, Airlangga University, Dr. Soetomo General Academic Hospital, Surabaya, East Java, Indonesia, unair.ac.id; ^2^ Pulmonology and Respiratory Medicine Department, Dr. Soetomo General Academic Hospital, Surabaya, East Java, Indonesia, rsudrsoetomo.jatimprov.go.id

## Abstract

Bilothorax, the presence of bile in the pleural cavity, is a rare and potentially life‐threatening condition. It may occur spontaneously or following hepatobiliary interventions, with right‐sided involvement being most common. Patients are at substantial risk of developing empyema and require prompt drainage and intravenous antibiotic therapy. We report a 41‐year‐old male presenting with progressive dyspnea and bilious pleural effusion. Imaging and pleural fluid analysis confirmed right‐sided bilothorax, supported by a pleural‐to‐serum bilirubin ratio > 1. Cultures later identified *Staphylococcus aureus*, indicating superimposed empyema. No prior hepatobiliary surgery was documented. Despite initial improvement with antibiotics and drainage, persistent pleural discharge and diaphragmatic injury warranted surgical intervention. Intraoperative findings revealed a 1 cm right hemidiaphragm defect with biliary contamination. Decortication and diaphragmatic repair using an abdominal wall fascial flap were performed. The patient showed clinical improvement postoperatively but declined follow‐up and later succumbed to sepsis. This case highlights the importance of early recognition, appropriate drainage, and timely surgical management for bilothorax complicated by empyema.

## 1. Introduction

Bilothorax refers to the presence of bile within the pleural cavity. The most common cause of bilothorax is iatrogenic injury, particularly involving damage to the biliary tract, which may result in the formation of a pleurobiliary fistula or a subphrenic abscess [1]. It represents an uncommon cause of exudative pleural effusion. Most reported cases occur on the right side, while bilateral involvement is unusual. Previous case reports have described the clinical presentation of bilothorax as acute abdominal pain accompanied by chills, followed by acute respiratory distress after biliary decompression [2, 3].

One known risk factor is a history of biliary peritonitis that was not adequately treated. Iatrogenic bilothorax is a common complication associated with percutaneous transhepatic biliary drainage (PTBD) and blunt abdominal trauma resulting in biliopleural fistula formation. Several mechanisms have been proposed to explain how bile enters the pleural space, including passive movement through the diaphragm, transport via lymphatic channels, traumatic or congenital diaphragmatic defects, and the formation of a biliary fistula [4, 5].

Bilothorax patients are more likely to develop empyema; thus, they need to be treated immediately with intravenous antibiotics and pleural space drainage to avoid infection. When infection spreads from other parts of the body to the pleural space, it results in thoracic empyema, which is the presence of purulent fluid in the pleural space. Gastrointestinal tract organisms such as *Escherichia coli*, *Enterobacter*, *Klebsiella oxytoca*, *Enterococcus faecalis*, and *Staphylococcus aureus* are the most frequent causes of complex and emphysematous bilious effusions [6–8].

## 2. Case Presentation

### 2.1. Initial Presentation and Patient′s History (November–December 2022)

A 41‐year‐old male was referred to the pulmonology clinic with a diagnosis of right‐sided bilothorax and bilateral pleural effusion, more prominent on the right. He had experienced progressively worsening dyspnea since November 2022, accompanied by decreased appetite and a 5 kg weight loss over 1 month. He also reported a brief episode of fever prior to initial hospitalization but denied cough, chest pain, night sweats, or gastrointestinal symptoms. He had a history of smoking 12 cigarettes per day since the age of 25, with a Brinkman index of 192, and had quit smoking 1 month prior to hospital admission. No immunosuppressive comorbidities were identified, including diabetes mellitus, chronic corticosteroid use, prior transplantation, chronic liver disease or cirrhosis, HIV/AIDS, or severe malnutrition.

**Figure 1:**
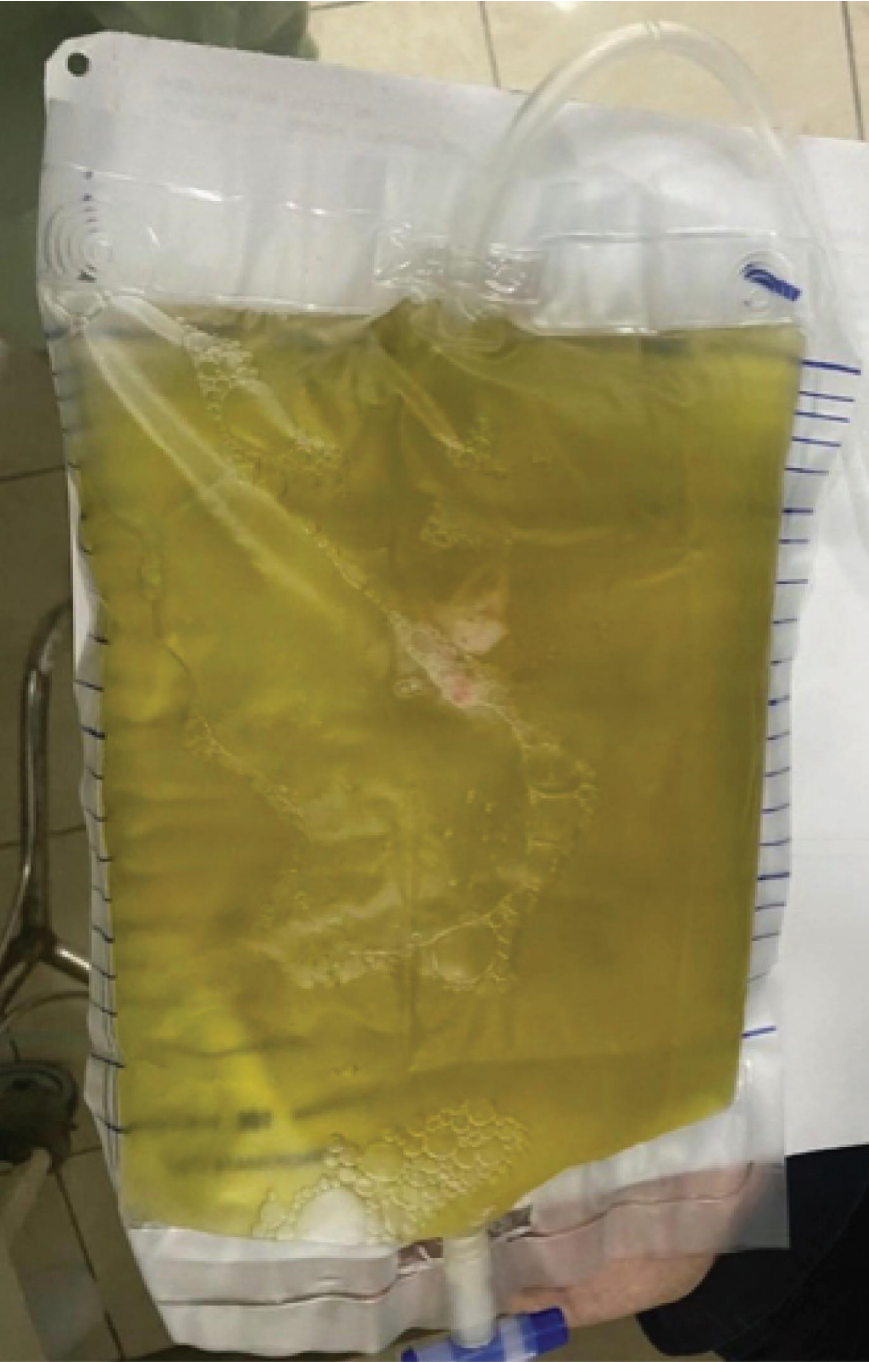
Greenish, bile‐stained pleural fluid obtained during thoracentesis from the right hemithorax, demonstrating the characteristic appearance of bilothorax.

His first hospitalization was in December 2022 with bilateral pleural effusion. Thoracentesis revealed 600 mL of greenish exudative pleural fluid (Figure 1). Given the subacute progressive dyspnea, recent 5 kg weight loss, and bilateral effusions in a high tuberculosis‐burden setting, empiric first‐line antituberculosis therapy was initiated while further investigations were arranged. However, his clinical condition worsened after 8 days, and the sputum GeneXpert was negative for *Mycobacterium tuberculosis*. Antituberculosis treatment was therefore discontinued, and alternative causes of the pleural effusion were investigated. Cytology of the pleural fluid was negative for malignancy.

He was rehospitalized in the same month with persistent dyspnea and intermittent fever. A diagnosis of right bilothorax was obtained from a pleural‐to‐serum bilirubin ratio > 1, with pleural fluid bilirubin (0.52 mg/dL) exceeding serum bilirubin (0.31 mg/dL). Fluid analysis showed an exudate with neutrophil predominance. He received levofloxacin and supportive medications. A total of 3150 mL of greenish fluid was drained from the right hemithorax and 500 mL of serous fluid from the left.

### 2.2. Diagnostic Evaluation and Interim Course (January–April 2023)

**Figure 2:**
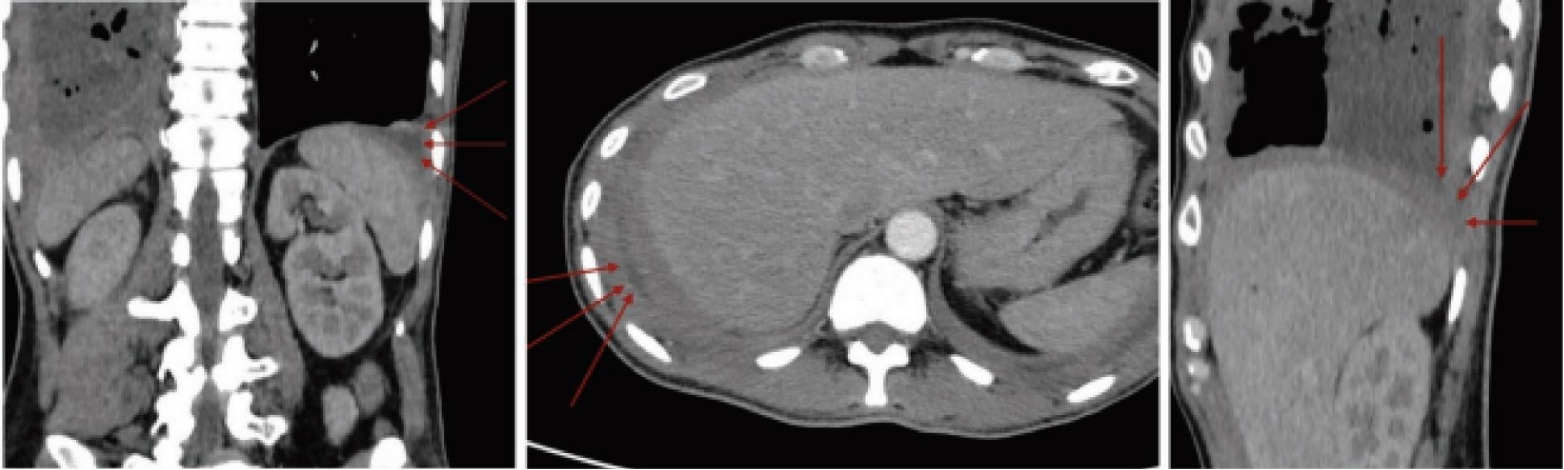
Contrast and noncontrast thoracic CT scan revealed bilateral pleural effusions, with compressive atelectasis of the right lower lobe and no evidence of pulmonary mass.

The patient underwent pigtail catheter insertion for a massive right pleural effusion. A contrast‐enhanced thoracic computed tomography (CT) scan in January 2023 revealed bilateral pleural effusions, compressive atelectasis of the right lower lobe, and no pulmonary mass (Figure 2). Additional findings included suspected tuberculomas, panacinar emphysema, and mediastinal lymphadenopathy. A multidisciplinary team meeting in February 2023 concluded that the etiology remained unclear, prompting a contrast abdominal CT scan in April 2023, which showed hepatomegaly but no intra‐abdominal or pelvic masses. While waiting for the abdominal CT scan, the patient did not return for follow‐up for 1 month, as he remained asymptomatic. At home, he reported continuous pleural drainage of 140–160 mL/day with yellowish‐green fluid.

### 2.3. Readmission With Catheter Dislodgement and Empyema (April 2023)

**Figure 3:**
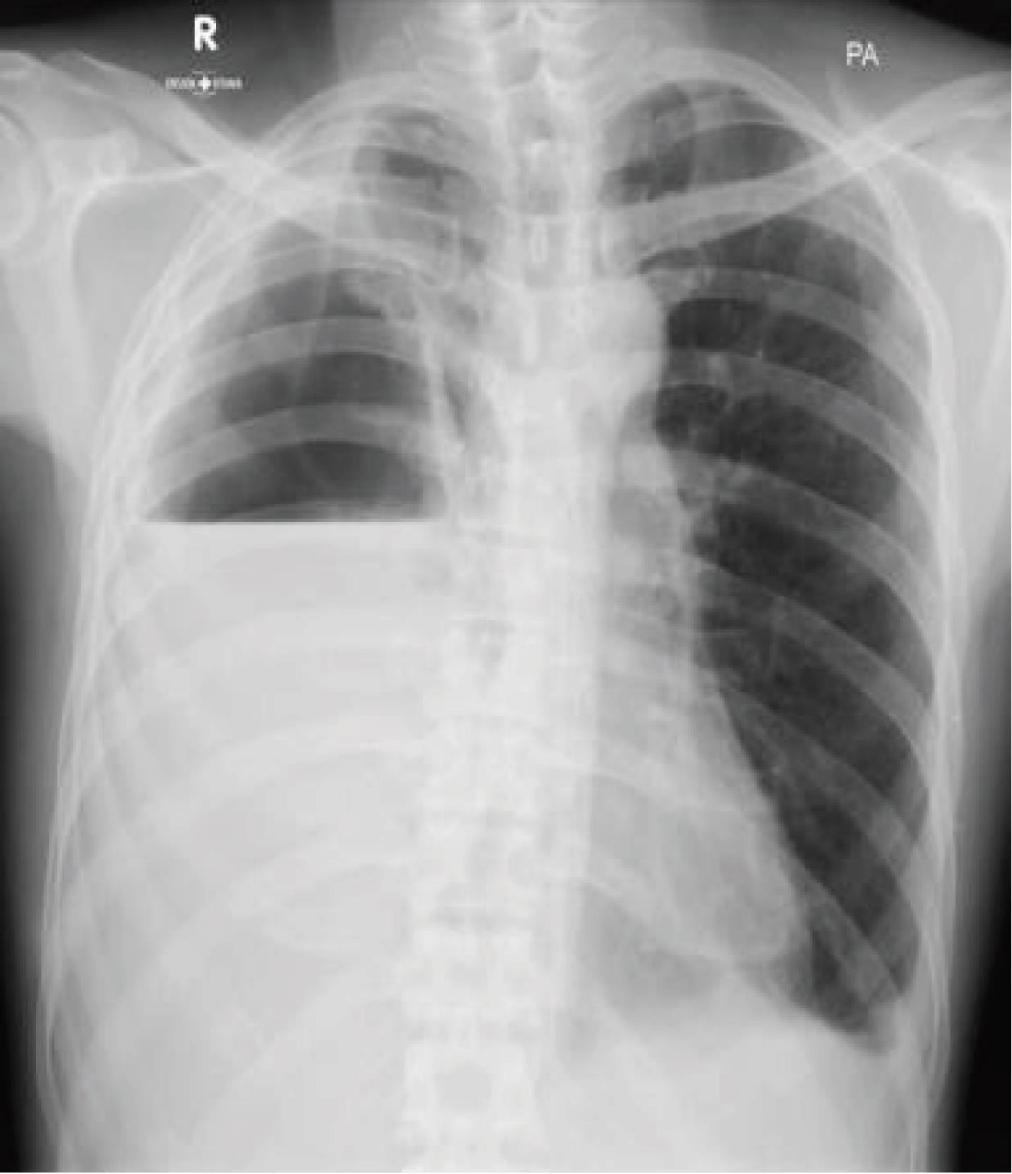
Chest x‐ray showing homogeneous opacity in the lower two‐thirds of the right hemithorax and an avascular area in the upper one‐third of the right hemithorax.

In April 2023, the patient came to the emergency department due to the dislodgement of the pigtail catheter. Chest examination revealed decreased expansion, fremitus, breath sounds, and dullness on percussion on the right. A chest tube was inserted, draining thick, white, foul‐smelling fluid, with an initial empyema output of 1200 mL on the first day. A chest radiograph revealed homogeneous opacities in the lower two‐thirds of the right and lower one‐third of the left hemithorax (Figure 3).

During hospitalization, the drainage volume gradually decreased. By the 10th day, daily output was approximately 100 mL, with the fluid becoming less viscous and clearer following administration of cefoperazone–sulbactam (2 g every 12 h) and metronidazole (500 mg every 8 h). The patient reported improvement in dyspnea after chest tube insertion, although he experienced chest tube–related pain and mild nausea without vomiting. Empyema culture grew *S. aureus*, while mycobacterial and fungal cultures were negative.

### 2.4. Surgical Intervention (April 2023)

The patient underwent multidisciplinary surgery. Surgical exploration by thoracic and digestive teams revealed a thick pleural peel covering all lobes of the right lung and diaphragm, with approximately 50 mL of yellowish pleural fluid (Figure 4a). Decortication was performed, and multiple fragments of necrotic pleural tissue were removed (Figure 4b). The lung re‐expanded > 80% with minimal leak. Although the diaphragm initially appeared intact, further exploration revealed a 1 cm defect in the right hemidiaphragm with surrounding tissue damage. Due to tissue fragility, the procedure was converted to open laparotomy, and the defect was repaired using an anterior abdominal wall fascia flap (Figures 5a, 5b, 5c, and 5d).

Figure 4Intraoperative findings. (a) Yellowish fluid accumulation on the right diaphragm, suggestive of biliary contamination. (b) Necrotic tissue removed during the surgery.

**Figure:**
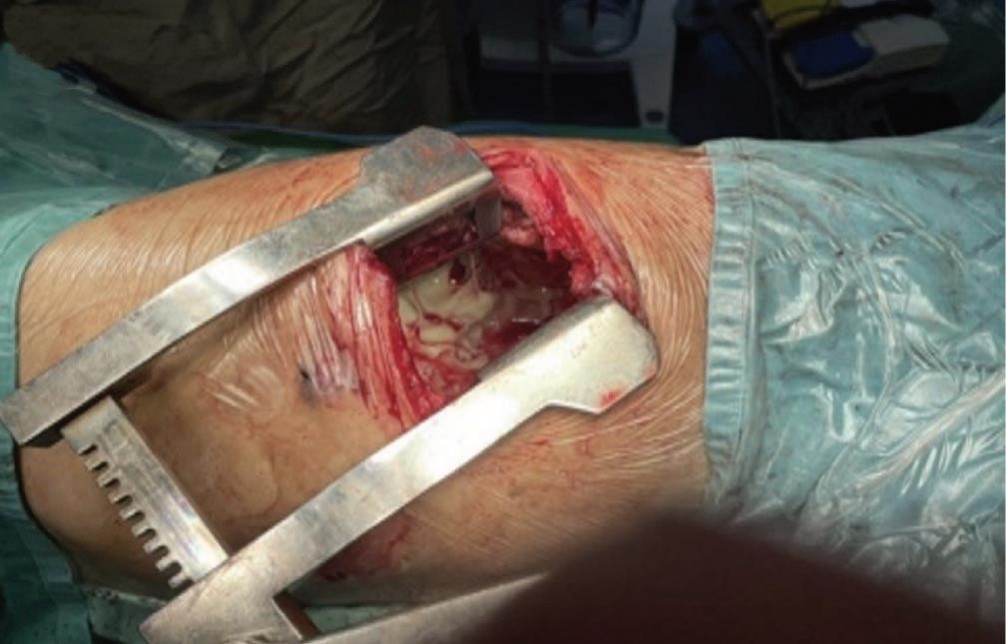
(a)

**Figure:**
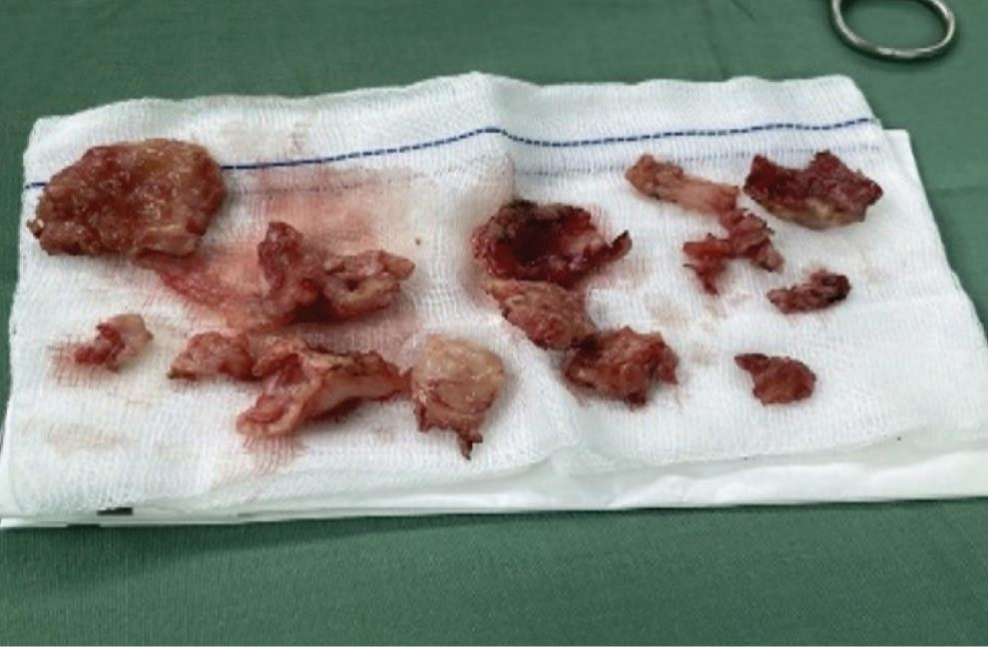
(b)

Figure 5Intraoperative findings observed during the surgical procedure on the patient. (a) Liver and right diaphragm. (b) Damage to liver tissue and the right diaphragm. (c) Defect in the right diaphragm. (d) Repair the diaphragm defect.

**Figure:**
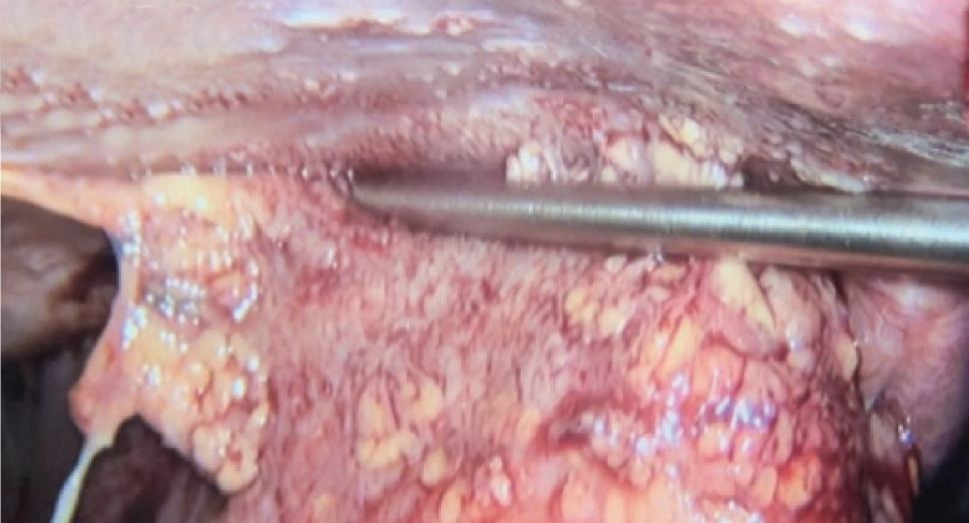
(a)

**Figure:**
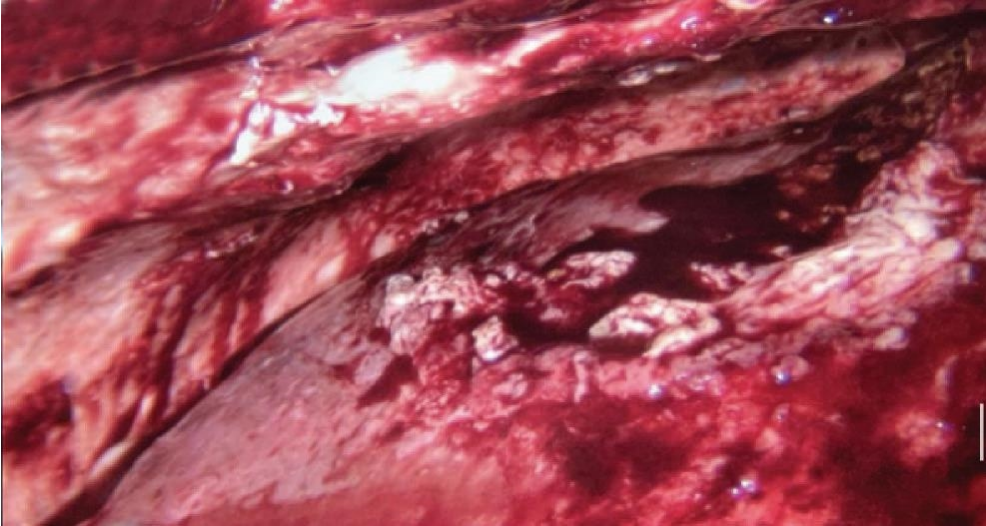
(b)

**Figure:**
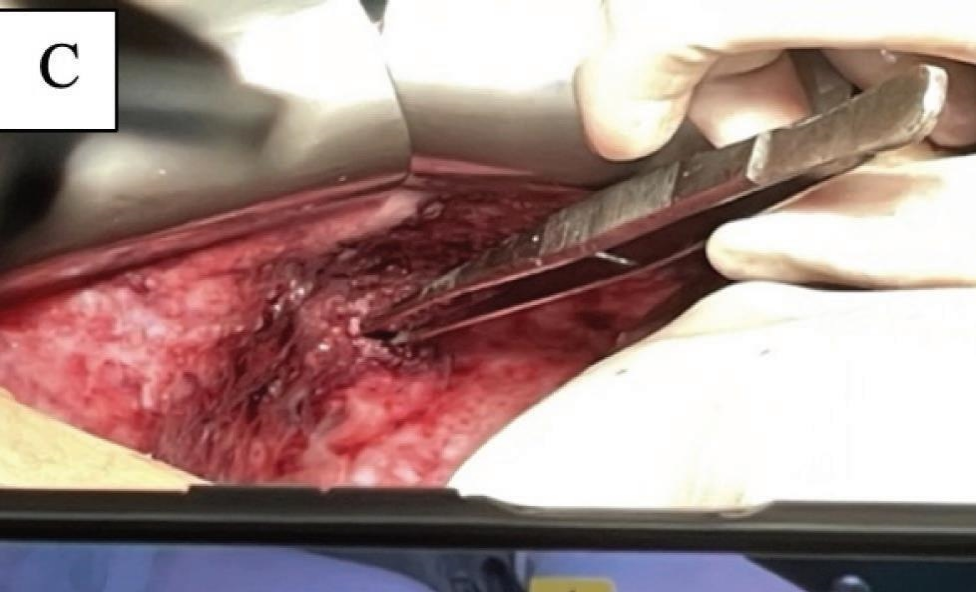
(c)

**Figure:**
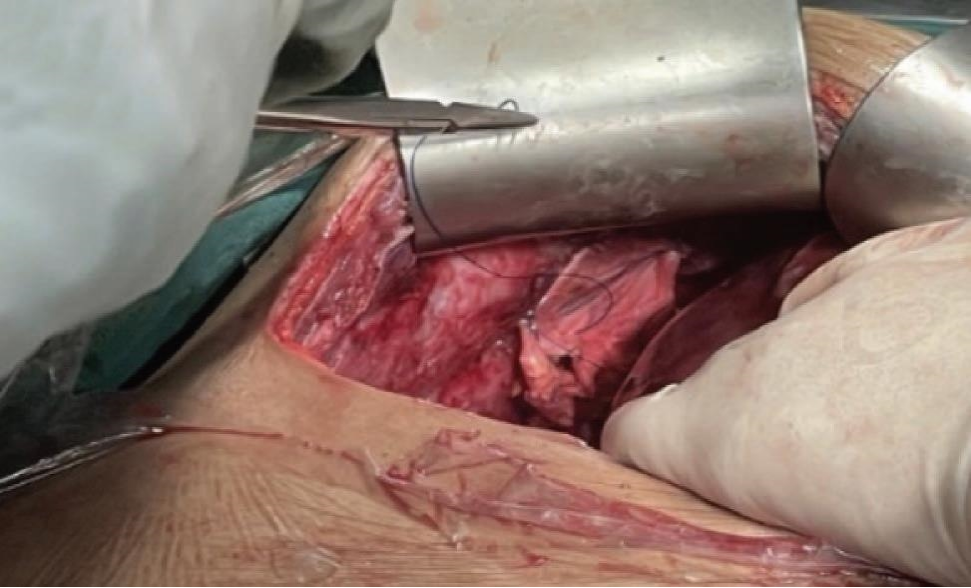
(d)

**Figure 6:**
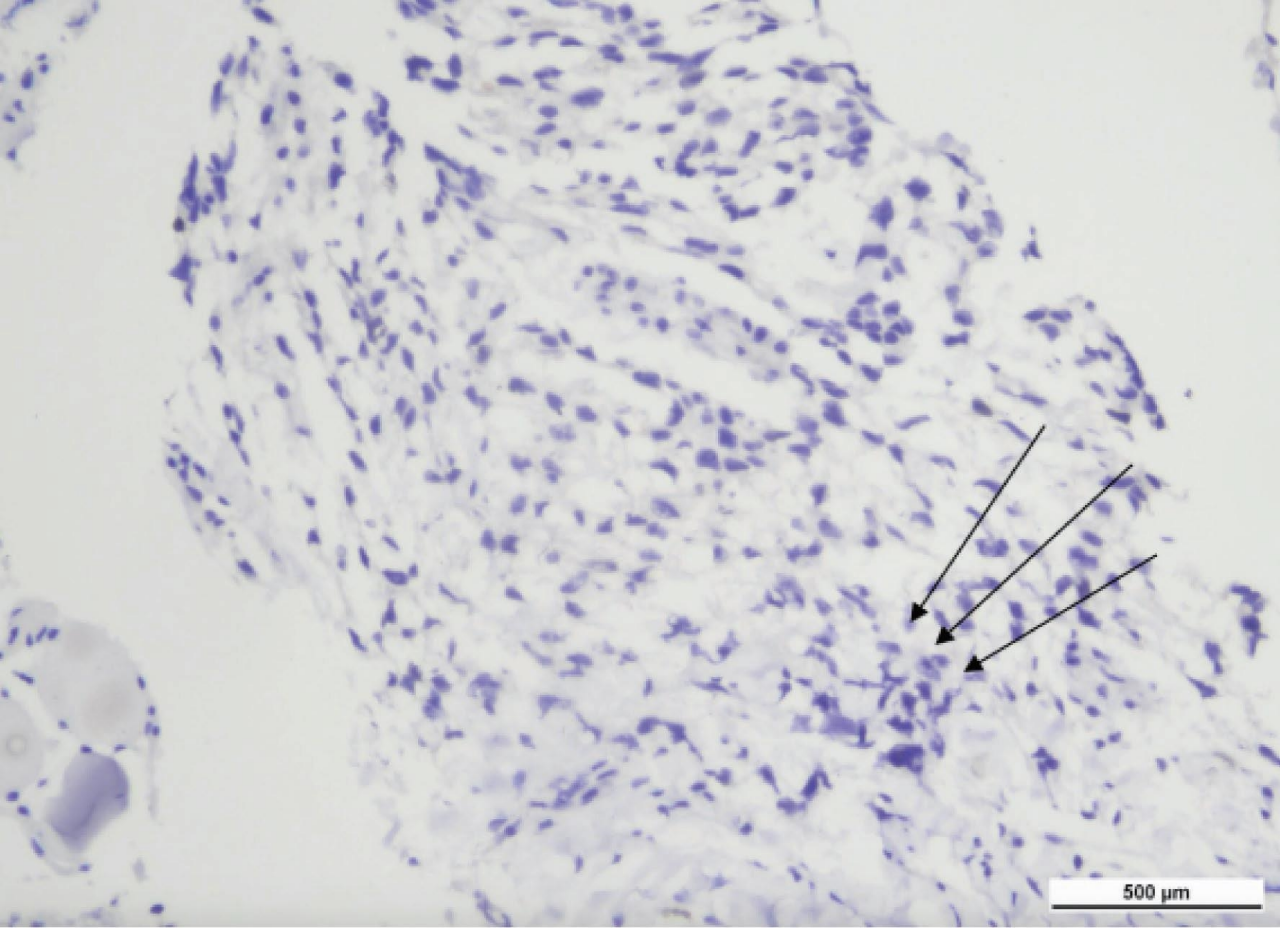
Histopathological examination of pleural tissue. Low‐power view showing fibrous pleural tissue with chronic inflammatory infiltrates (hematoxylin and eosin stain; scale bar 500 *μ*m).

Pleural tissue removed during decortication was sent for histopathological examination. The specimen demonstrated fibrous pleural tissue with dense mixed inflammatory infiltrates of lymphocytes, neutrophils, and histiocytes, in keeping with chronic suppurative pleuritis, with no evidence of malignancy or granulomatous inflammation (Figure 6).

A chest tube and an indwelling pleural catheter were inserted. On admission with empyema, the patient received empirical intravenous cefoperazone–sulbactam and metronidazole for 14 days. During this period, particularly after thoracotomy, decortication, and surgical debridement, the pleural drainage became less purulent and his respiratory symptoms improved. Once the pleural fluid culture grew *S. aureus* susceptible to amikacin, the antibiotic regimen was de‐escalated to intravenous amikacin alone as continuation therapy for a further 7 days, according to the susceptibility profile and local protocol. Clinical improvement was observed within 3 weeks: reduced cough, no dyspnea or fever, and decreased pleural output. The chest tube and IPC were removed following radiological and clinical stability.

### 2.5. Outcome and Final Readmission (April–September 2023)

By the fourth week postsurgery, the patient had no respiratory complaints, tolerated oral intake, and was discharged for outpatient follow‐up with the pulmonology and surgical teams. According to his wife, the patient declined further hospital follow‐up, as he felt clinically improved and believed no further treatment was necessary. Thus, he did not attend scheduled wound‐care follow‐ups, which likely led to inadequate postoperative healing and subsequent secondary infection.

In September 2023, he was readmitted due to severe clinical deterioration with clinical features consistent with sepsis. Given his history of *S. aureus* empyema and suboptimal wound and catheter‐site care, recurrent or persistent thoracic infection was considered the most likely source, although no repeat cultures were obtained during this short admission. He was hospitalized for 2 days and died with a diagnosis of infection accompanied by poor oral intake.

## 3. Discussion

Bilothorax is a rare condition defined as the presence of bile within the pleural cavity, most commonly affecting the right hemithorax. Due to its low incidence, epidemiological data are limited. A review by Austin et al. identified only 59 reported cases between 1960 and 2017, with 13 of them associated with PTBD. Similarly, Karnik and Shair reported 14 cases, of which four were PTBD‐related, corresponding to an estimated prevalence of 0.5% of all PTBD procedures. The condition is considered a medical emergency because of the inflammatory and cytotoxic nature of bile, which can rapidly lead to empyema and other complications if not promptly treated [4–11].

The etiology of bilothorax is diverse, but iatrogenic causes such as prior hepatobiliary procedures—especially PTBD [4, 5], liver transplantation [9], and hepatic resections [10]—are among the most commonly reported. Other causes include trauma to the diaphragm, subphrenic infections or abscesses, obstructive biliary disease, tuberculosis, and, rarely, idiopathic mechanisms [1, 11–13]. In this patient, no history of prior hepatobiliary surgery was documented, and the patient presented without jaundice or right upper quadrant abdominal pain. However, imaging revealed hepatic abnormalities, and intraoperative findings confirmed a diaphragmatic defect and localized bile in the pleural cavity. These findings are compatible with a spontaneous pleurobiliary communication across the diaphragm. However, because the hepatic tissue was not submitted for histopathological or microbiological analysis, the exact intra‐abdominal source of the biliary leak could not be definitively identified.

The pathogenesis of bilothorax involves either direct or indirect entry of bile into the pleural space. One mechanism suggests passive transdiaphragmatic migration of bile through anatomical pores, similar to the process in hepatic hydrothorax, especially when negative intrathoracic pressure facilitates the movement. Another mechanism proposes lymphatic spread via pleuroperitoneal lymphatic channels. Importantly, bile itself can cause direct chemical injury to surrounding tissues, leading to necrosis and fistula formation even without surgical trauma [ 10, 11, 14, 15]. In this case, the surgical discovery of a 1 cm diaphragmatic defect and inflammatory adhesion between the diaphragm, liver, and omentum suggests both mechanical disruption and chemical damage played a role.

Microbial infection is a critical concern in bilothorax, as bile stasis provides an excellent medium for bacterial growth. The most commonly implicated organisms include *E. coli*, *Klebsiella pneumoniae*, *E. faecalis*, and *S. aureus* [15, 16]. In our patient, the isolation of *S. aureus* from the pleural fluid in the setting of a long‐term indwelling pleural catheter makes catheter‐associated infection a likely source of the empyema. Indwelling pleural catheters are increasingly used for recurrent pleural effusions, and pleural infection occurs in about 5%–6% of patients, with *S. aureus* being the most frequently reported pathogen in large series and recent reviews [17–19]. Prolonged catheter dwell time may facilitate biofilm formation and deep pleural space infection, underscoring the need for meticulous catheter care, regular surveillance, and timely reassessment of the indication for ongoing drainage.

This patient presented with purulent, foul‐smelling pleural fluid consistent with empyema, which responded to broad‐spectrum antibiotics including cefoperazone–sulbactam and metronidazole. Although tuberculosis was considered due to radiological features and chronic weight loss, no acid‐fast bacilli were detected via GeneXpert, culture, or histopathology. The patient′s clinical improvement without antituberculous therapy further excluded TB as the causative agent.

Diagnosis of bilothorax relies on a combination of clinical, radiological, and laboratory data. Patients typically present with respiratory symptoms such as dyspnea and pleuritic chest pain. In our case, the patient reported shortness of breath without abdominal complaints. Radiographs may reveal right‐sided pleural effusion, as seen in this patient′s chest x‐ray, which demonstrated homogenous opacity in the lower hemithorax. CT imaging aids in identifying hepatic pathology and possible diaphragmatic defects. The definitive diagnosis is made via pleural fluid analysis, where a pleural‐to‐serum bilirubin ratio > 1 is pathognomonic, as it indicates that the bilirubin concentration in pleural fluid exceeds that of serum, confirming the presence of bile within the pleural space [3, 15]. In this case, pleural fluid bilirubin was 0.52 mg/dL and serum bilirubin was 0.31 mg/dL, with a pH of 7.0—supporting the diagnosis.

Management of bilothorax is guided by the underlying etiology and severity. Most reports advocate initial conservative treatment, including adequate pleural drainage and empirical antibiotic coverage to prevent empyema [20]. In this case, initial chest tube drainage produced over 1200 mL of purulent bile, which progressively decreased following antibiotic therapy. Definitive surgical management was required due to persistent drainage and suspected diaphragmatic injury. The patient underwent thoracotomy, decortication, and diaphragmatic repair with an abdominal wall fascial flap. This approach aligns with prior case series advocating early surgical intervention in complicated bilothorax cases, such as patients with diaphragmatic trauma and pleurobiliary fistula [1, 8, 21].

Our antibiotic strategy, therefore, consisted of an initial 14‐day course of broad‐spectrum *β*‐lactam–based therapy followed by short amikacin monotherapy as de‐escalation once clinical improvement had occurred after surgical source control. We acknowledge that this approach differs from guideline‐preferred antistaphylococcal regimens for *S. aureus* empyema and should be regarded as a limitation of the present case, rather than a recommended standard of care.

Despite successful surgical repair and clinical improvement, the patient discontinued follow‐up because he felt asymptomatic and believed no further treatment was necessary. This interruption in care resulted in the absence of wound monitoring, which likely contributed to the development of a new infectious process. Without scheduled evaluation, early signs of infection, nutritional decline, or recurrent pleural contamination went unrecognized. By the time the patient returned to the hospital, he had already developed significant clinical deterioration. This highlights the importance of close postdischarge monitoring in cases of complex thoracoabdominal pathology, where complications may recur despite initial improvement. Timely diagnosis and comprehensive management—medical, surgical, and supportive—are crucial for favorable outcomes in this rare but potentially life‐threatening condition.

## 4. Conclusion

This case highlights the complexity of managing bilothorax complicated by empyema when the underlying etiology remains unclear. Early recognition, adequate drainage, and surgical repair facilitated initial improvement, but the patient′s loss to follow‐up contributed to delayed identification of recurrent infection and eventual fatal deterioration. This underscores the importance of early recognition, multidisciplinary management, and strict postdischarge follow‐up in optimizing outcomes for patients with bilothorax.

## Funding

No funding was received for this manuscript.

## Consent

Patient consent was obtained.

## Conflicts of Interest

The authors declare no conflicts of interest.

## Data Availability

Data sharing is not applicable to this article as no datasets were generated or analyzed during the current study.
